# The effect of vitamin D on adolescents’ primary dysmenorrhea

**DOI:** 10.25122/jml-2023-0290

**Published:** 2023-11

**Authors:** Ainur Donayeva, Ainur Amanzholkyzy, Ibrahim Abdelazim, Meirambek Kurmangazin, Zaituna Khamidullina, Madina Kurmanalina, Aigul Sumanova, Zhanara Shabanbayeva, Zhenisbek Baubekov, Bauyrzhan Bissaliyev, Gulnara Gubasheva, Ihab Samaha

**Affiliations:** 1 Department of Normal Physiology, West Kazakhstan Marat Ospanov Medical University, Aktobe, Kazakhstan; 2 Department of Obstetrics and Gynecology, Faculty of Medicine, Ain Shams University, Cairo, Egypt; 3 Department of Obstetrics and Gynecology №^1^, Astana Medical University, Astana, Kazakhstan; 4 Department of Therapeutic and Orthopedic Dentistry, West Kazakhstan Marat Ospanov Medical University, Aktobe, Kazakhstan; 5 Department of Pediatric Surgery, West Kazakhstan Marat Ospanov Medical University, Aktobe, Kazakhstan; 6 Department of Obstetrics and Gynecology №^2^, West Kazakhstan Marat Ospanov Medical University, Aktobe, Kazakhstan; 7 Department of Obstetrics and Gynecology, Faculty of Medicine, Helwan University, Cairo, Egypt

**Keywords:** adolescents, dysmenorrhea, vitamin D, 25(OH)D: 25-Hydroxyvitamin D, ADA: American Diabetes Association, ASRM: American Society for Reproductive Medicine, BMI: Body Mass Index, ESHRE: European Society of Human Reproduction and Embryology, HbA1C: Glycosylated Hemoglobin, NSAIDs: Nonsteroidal Anti-Inflammatory Drugs, PCOS: Polycystic Ovary Syndrome, PGDs: Prostaglandins, QoL: Quality of Life, TSH: Thyroid-Stimulating Hormone, VAS: Visual Analog Scale, VDR: Vitamin D Receptor, Vit. D: Vitamin D, VMS: Verbal Multidimensional Scoring System, WKU: West Kazakhstan University

## Abstract

Vitamin D receptor (VDR) expression in the female reproductive tract explains the regulatory role of vitamin D on inflammatory cytokine and prostaglandin (PGD) synthesis. This study aimed to evaluate the effect of vitamin D on adolescents’ primary dysmenorrhea and the relationship between Vit. D and adolescents’ primary dysmenorrhea. Eighty-five adolescents were included in the current study. After a detailed evaluation, pelvic sonography was performed for all participants to rule out any pelvic pathology. Blood samples were collected to measure thyroid stimulating hormone (TSH), prolactin, glycosylated hemoglobin (HbA1C), and 25-hydroxyvitamin D (25[OH]D). Participants were administered vitamin D (50,000 IU weekly for five months), and their dysmenorrhea symptoms were evaluated before and after this period using the Visual Analog Scale (VAS) and the Verbal Multidimensional Scoring (VMS). The mean VAS and VMS scores of dysmenorrhea statistically decreased from 8.7±0.91 and 2.65±0.93 to 4.8±0.75 and 0.80±0.75, respectively, after vitamin D intake (p=0.03 and 0.025, respectively). Significant negative associations between 25(OH)D and VAS (R = -0.886; p<0.00001) and VMS of dysmenorrhea (R = -0.885; p<0.00001) were detected in this study. Vit. D could be a useful therapeutic option to reduce the severity of primary dysmenorrhea and could limit the use of non-steroidal anti-inflammatory drugs.

## INTRODUCTION

Dysmenorrhea, characterized by painful menstrual periods, is a common condition that can include additional symptoms such as nausea, sleep disturbances, and mood changes [1, 2]. It is a prevalent issue, impacting between 16% and 91% of women of childbearing age and around 80% of teenagers [1, 2]. There are two types of dysmenorrhea: primary, which occurs without any underlying pelvic conditions, and secondary, often linked to pelvic disorders like endometriosis or adenomyosis [1-3]. The underlying mechanisms of dysmenorrhea are believed to involve an increase in the production of substances like prostaglandins (PGD) and leukotrienes in the uterus, which can lead to heightened uterine contractions and reduced blood flow [4-5]. This condition can negatively affect the quality of life [6].

The standard treatment for dysmenorrhea includes nonsteroidal anti-inflammatory drugs (NSAIDs) and oral contraceptives [7]. NSAIDs decrease the severity of dysmenorrhea by inhibiting PGD synthesis, but they increase the risk of gastrointestinal bleeding and gastric ulcers [8]. There is little evidence regarding the efficacy of oral contraceptives in the treatment of dysmenorrhea, and 50% of women discontinue their use due to adverse side effects [9]. Exploring alternative treatments for dysmenorrhea could reduce reliance on NSAIDs and oral contraceptives, offering safer and more effective management options. The presence of Vitamin D receptors in the female reproductive system suggests that Vitamin D could influence the synthesis of inflammatory cytokines and prostaglandins [10-12]. A systematic review found an inverse relationship between the severity of dysmenorrhea and vitamin D [13]. Karacin *et al*. [14] reported a negative correlation between severe dysmenorrhea and vitamin D. Additionally, Kucukceran *et al*. [15] found that menstrual pain and the consumption of NSAIDs were significantly reduced after a single dose of oral cholecalciferol compared to placebo.

Given the hypothesized impact of Vitamin D on inflammatory cytokines and PGD synthesis, this study aims to investigate the impact of Vitamin D on primary dysmenorrhea in adolescents, exploring the potential relationship between Vitamin D levels and the severity of this condition.

## MATERIAL AND METHODS

### Study design

A total of 85 adolescents were included in the current prospective cohort study conducted in the Republic of Kazakhstan from 2021 to 2022 to evaluate the effect of Vit. D on adolescents’ primary dysmenorrhea.

### Participants

Eighty-five adolescents were enrolled. Participants were selected based on inclusion criteria: girls aged >12 and <18 years with regular menstrual cycles, normal BMI (18.5-24.9 kg/m^2^), experiencing primary dysmenorrhea for ≥ six months and having vitamin D deficiency. Exclusion criteria were age outside the specified range, BMI extremes (underweight, overweight, obese), irregular menstrual cycles, existing medical or endocrine disorders, prior steroid hormone or vitamin D supplementation, and non-consent.

### Assessment

Each participant underwent a thorough evaluation process. This included a detailed medical history review, a comprehensive clinical examination, the calculation of BMI, and pelvic sonography to rule out any pelvic abnormalities. Primary dysmenorrhea, the focus of this study, is characterized by painful menstrual cramps that typically start just before and may continue for a short duration after the onset of menstrual flow. While primary dysmenorrhea is not linked to pelvic abnormalities or lesions, it can be accompanied by other symptoms such as vomiting, insomnia, and mood changes [1, 2]. The diagnosis of polycystic ovary syndrome was based on the criteria established by the European Society of Human Reproduction and Embryology and the American Society for Reproductive Medicine [16, 17]. Diabetes was defined according to the American Diabetic Association as a metabolic disorder characterized by hyperglycemia (either from insulin deficiency or defective insulin action) and diagnosed when glycosylated hemoglobin (HbA1C) and fasting plasma glucose were ≥ 6.5% and ≥ 126 mg/dL, respectively [18]. Hypertension was diagnosed when the systolic and/or diastolic blood pressure were ≥ 140 mmHg and/or ≥ 90 mmHg, respectively (on two different occasions) [19].

### Laboratory measurements

Blood samples were collected from the adolescents to measure key health indicators: thyroid-stimulating hormone (TSH), prolactin, glycosylated hemoglobin (HbA1C), and 25-hydroxyvitamin D (25(OH)D). The normal ranges for these indicators were established as follows: serum TSH between 0.4-4.0 mIU/mL [20], serum prolactin below 29 ng/mL [21], and HbA1C under 6.5% [14]. Serum 25(OH)D levels were used as a reliable marker for vitamin D status, with levels above 30 ng/mL indicating normal vitamin D and below 20 ng/mL indicating deficiency [22]. Blood samples were centrifuged and stored at -20 °C for quantitative 25(OH)D evaluation. The 25(OH)D level was measured using Architect (Abbott, Longford, Ireland), a delayed one-step chemiluminescence microparticle method, using the Architect 25(OH)D Reagent Kit [23].

### Vitamin D administration and follow-up

In this study, participants were administered vitamin D at a dosage of 50,000 international units (IU) weekly for five months, following the protocol established by the hospital. The effectiveness of this vitamin D supplementation was assessed in the sixth month. This evaluation involved comparing the levels of 25(OH)D in the participants, as well as their scores on the Visual Analog Scale (VAS) and the Verbal Multidimensional Scoring (VMS) scales before and after the supplementation period. The primary objective of this assessment was to determine the efficacy of Vitamin D in managing primary dysmenorrhea among adolescent participants. A secondary objective involved examining the correlation between vitamin D levels and dysmenorrhea severity using Pearson's correlation analysis. The VAS, a 10-point scale (0=no pain, 10=worst pain) [24], and the VMS, a 4-point scale (ranging from no pain to severe pain) [25], were employed to evaluate dysmenorrhea severity.

### Statistical analysis

G Power 3.1.9.7 was used to calculate the sample size with a 0.05 probability, 0.95% power, and an effect size of 0.5 [25, 26]. The Student’s t-test and Pearson correlation analysis were used to assess relationships between variables. A p<0.05 was considered significant.

## RESULTS

This was conducted between 2021 and 2022, included 85 adolescents from Kazakhstan, and assessed the impact of Vitamin D (50,000 IU weekly for 5 months) on primary dysmenorrhea. Severity was measured using the VAS and the VMS scales.

**Table 1:** Baseline characteristics and initial assessment scores

Variables	Participants (N=85 adolescents)
Age (years)	14.82±1.7
Weight (kg)	58.41±4.2
Height (cm)	158.52±2.8
BMI (kg/m^2^)	23.22±1.32
25(OH)D (ng/mL)	13.5±2.9
VAS	8.7±0.91
VMS	2.65±0.93

25(OH)D: 25-Hydroxyvitamin D. BMI: Body mass index

Data presented as mean ± SD (standard deviation).

VAS: Visaual Analogue scale.

VMS: Verbal Multidimensional Scoring System.

**Figure 1:**
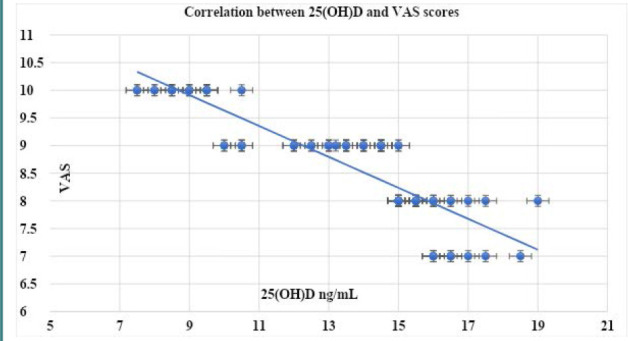
Correlation between the 25(OH)D and VAS scores VAS: Visual analogue scale.

**Figure 2:**
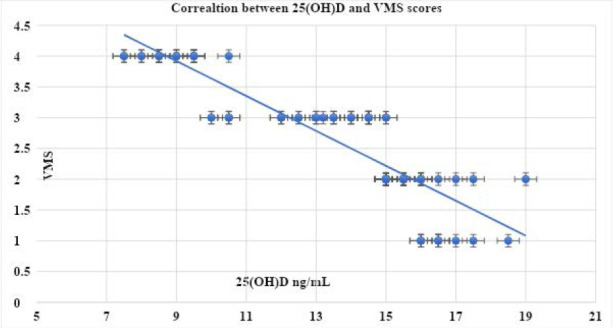
Correlation between the 25(OH)D and VMS scores VMS: Verbal Multidimensional Scoring System.

The characteristics of the participants are described in Table 1. The initial mean 25(OH)D level of participants was 13.5±2.9 ng/mL. The mean baseline VAS and VMS scores were 8.7±0.91 and 2.65±0.93, respectively (Table 1). The mean 25(OH)D significantly increased to 58.4±2.3 ng/mL (p=0.01; 95% CI: -45.7, -44.9, -44.11) after vitamin D intake. In addition, there was a significant decrease in the mean VAS and VMS scores (4.8±0.75 and 0.80±0.75, respectively) after Vit. D intake (p=0.03; 95% CI: 3.65, 3.9, 4.153, and 0.025; 95% CI: 1.6, 1.85, 2.11, respectively) (Table 2). There was a significant negative association between 25(OH)D and VAS (r -0.886; p<0.00001) (Figure 1) and VMS scores (r -0.885; p<0.00001) (Figure 2).

**Table 2:** Comparative analysis of vitamin D levels and dysmenorrhea severity scores before and after vitamin D intake

Variables	Before Vit. D intake (N=85 adolescents)	After Vit. D intake (N=85 adolescents)	p-value (95% CI)
25(OH)D (ng/mL)	13.5±2.9	58.4±2.3	0.01* (-45.7, -44.9, -44.11)
VAS	8.7±0.91	4.8±0.75	0.03* (3.65, 3.9, 4.153)
VMS	2.65±0.93	0.80±0.75	0.025* (1.6, 1.85, 2.11)

* : Significant difference. 25(OH)D: 25-hydroxy Vit. D. CI: Confidence Interval. Data presented as mean ± SD (standard deviation). Student’s t-test used for statistical analysis. VAS: Visual Analogue Scale. VMS: Verbal Multidimensional Scoring System.

## DISCUSSION

Dysmenorrhea affects 16% to 91% of reproductive-age women and 80% of adolescents [1, 2]. The treatment of dysmenorrhea with therapeutic options other than NSAIDs and oral contraceptives could be helpful and limit the use of NSAIDs and oral contraceptives. VDR expression in the female reproductive tract [10] explains the regulatory role of Vit. D on inflammatory cytokine and PGD synthesis [11, 12]. Serum 25(OH)D is an accurate predictor of the actual Vit. D status [22].

In our study, 85 adolescents received vitamin D supplementation (50,000 IU weekly for 5 months) according to the hospital`s protocol. We observed significant increases in serum 25(OH)D levels (from 13.5±2.9 ng/mL to 58.4±2.3 ng/mL, p=0.01) and notable decreases in both VAS and VMS scores of dysmenorrhea (p=0.03 and 0.025, respectively). In addition, we identified significant negative associations between 25(OH)D and both VAS (p<0.00001) and VMS scores (p<0.00001) in this study.

The results of this study align with findings from other research, underscoring the potential role of Vitamin D in managing dysmenorrhea. An observational trial identified that participants with lower serum Vitamin D levels experienced more severe dysmenorrhea [27]. A randomized controlled study reported lower serum Vit. D in dysmenorrhea with a negative correlation between dysmenorrhea and Vit. D [14]. A randomized comparative trial found that the severity of dysmenorrhea and NSAID use was significantly reduced after a 300,000 IU cholecalciferol single dose compared to placebo [15]. Another randomized controlled study found that Vit. D intake significantly reduced the severity of dysmenorrhea and the amount of consumed analgesics [3]. Bahrami *et al*. [28] demonstrated that a high intake of cholecalciferol (50,000 IU weekly for nine weeks) significantly reduced dysmenorrhea severity. Similarly, Amzajerdi *et al*. [25] reported that high-dose Vitamin D supplementation (300,000 IU/day) significantly decreased the VAS and VMS scores for dysmenorrhea over two months. A systematic review reported significantly reduced dysmenorrhea after vitamin D and calcium supplementation [13].

The exclusion of underweight, overweight, or obese adolescents from this study was justified by the unclear relationship between dysmenorrhea and BMI in existing research [29]. A longitudinal study [30] found an increased risk of dysmenorrhea in women who were either underweight or obese. Similarly, Jiang *et al*. [31] reported increased severity of dysmenorrhea in participants with lower or higher BMIs. The controversial relationship between BMI and dysmenorrhea needs further study.

This study was the first to evaluate the effect of Vit. D (50,000 IU weekly for five months) on adolescents’ primary dysmenorrhea and the relationship between Vit. D and adolescents’ primary dysmenorrhea in the Republic of Kazakhstan.

Some of the limitations of this study are the refusal of some adolescents to consent and a relatively short-term follow-up period. The effect of Vit. D (50,000 IU weekly for five months) on the severity of primary dysmenorrhea must be confirmed in more extensive studies.

## CONCLUSION

Vitamin D could be a useful therapeutic alternative to reduce the severity of primary dysmenorrhea and could limit the use of NSAIDs. The effect of vitamin D on the severity of primary dysmenorrhea needs to be confirmed in further larger studies.
